# Epidermal-Derived Hedgehog Signaling Drives Mesenchymal Proliferation during Digit Tip Regeneration

**DOI:** 10.3390/jcm10184261

**Published:** 2021-09-20

**Authors:** Zeshaan N. Maan, Yuval Rinkevich, Janos Barrera, Kellen Chen, Dominic Henn, Deshka Foster, Clark Andrew Bonham, Jagannath Padmanabhan, Dharshan Sivaraj, Dominik Duscher, Michael Hu, Kelley Yan, Michael Januszyk, Michael T. Longaker, Irving L. Weissman, Geoffrey C. Gurtner

**Affiliations:** 1 Department of Surgery, Division of Plastic and Reconstructive Surgery, Stanford University School of Medicine, Stanford, CA 94305, USA; zmaan@stanford.edu (Z.N.M.); janosb@stanford.edu (J.B.); kellenchen@stanford.edu (K.C.); dhenn2@stanford.edu (D.H.); dsfoster@stanford.edu (D.F.); cbonham@stanford.edu (C.A.B.); jaganpa@stanford.edu (J.P.); ds311@stanford.edu (D.S.); dominikduscher@me.com (D.D.); hums2@upmc.edu (M.H.); januszyk@stanford.edu (M.J.); longaker@stanford.edu (M.T.L.); 2 Institute for Stem Cell Biology and Regenerative Medicine, Stanford University, Stanford, CA 94305, USA; irv@stanford.edu; 3 Helmholtz Zentrum München, Institute of Regenerative Biology & Medicine, 81377 Munich, Germany; 4 Department of Plastic, Reconstructive, Hand and Burn Surgery, BG-Trauma Center, Eberhard Karls University Tübingen, 72074 Tübingen, Germany; 5 Department of Medicine, Division of Digestive and Liver Diseases, Columbia University Medical Center, New York, NY 10032, USA; ky2004@cumc.columbia.edu

**Keywords:** digit tip, regeneration, hedgehog signaling, sonic hedgehog, Wnt, rainbow mouse, epimorphic regeneration, clonal proliferation, germ layer

## Abstract

Hand injuries often result in significant functional impairments and are rarely completely restored. The spontaneous regeneration of injured appendages, which occurs in salamanders and newts, for example, has been reported in human fingertips after distal amputation, but this type of regeneration is rare in mammals and is incompletely understood. Here, we study fingertip regeneration by amputating murine digit tips, either distally to initiate regeneration, or proximally, causing fibrosis. Using an unbiased microarray analysis, we found that digit tip regeneration is significantly associated with hair follicle differentiation, Wnt, and sonic hedgehog (SHH) signaling pathways. Viral over-expression and genetic knockouts showed the functional significance of these pathways during regeneration. Using transgenic reporter mice, we demonstrated that, while both canonical Wnt and HH signaling were limited to epidermal tissues, downstream hedgehog signaling (through Gli) occurred in mesenchymal tissues. These findings reveal a mechanism for epidermal/mesenchyme interactions, governed by canonical hedgehog signaling, during digit regeneration. Further research into these pathways could lead to improved therapeutic outcomes after hand injuries in humans.

## 1. Introduction

The human hand is a powerful tool for interaction with the environment. Its complex anatomy and mechanics allow it to perform a variety of functions, including labor, sensation, and communication [1,2,3]. Amputation injuries affecting the hand and upper extremity can lead to severe functional impairment and affect almost every aspect of a person’s life. Even with expert surgical intervention and rigorous occupational therapy, it has proven challenging to fully restore pre-injury hand function due to the minimal regenerative capacity of the various tissue types that constitute the hand [1,2]. In humans and most mammals, the tissue repair process is characterized by excessive fibrosis, resulting in the replacement of limb tissue with a dysfunctional patch of cells and disorganized extracellular matrix, commonly referred to as scar tissue [4,5]. The fibrotic “patch” repair process restores the barrier between the body and the external environment but is largely devoid of native tissue properties. In a part of the body reliant on harmonious anatomic interplay, this leads to unsatisfactory functional outcomes [1,4,6]. 

Some species of animals, such as salamanders, frogs, and zebrafish, can naturally regenerate limbs after an amputation injury [1,4]. However, the regeneration of limb tissue in mammals is extremely limited, with the injury response primarily dominated by fibrosis. An improved understanding of the molecular mechanisms that mediate the switch from “regenerative healing” to “fibrotic healing” could provide new opportunities for treating patients with hand injuries. In this regard, the rare instances of mammalian tissue regeneration merit further scrutiny. In young children, a strong regenerative capacity has been documented following accidental amputations of the finger proximal to the nail but distal to the distal interphalangeal joint [7,8]. Similarly, mice have been shown to spontaneously regenerate their digit tips following amputations just below the nail [9,10]. A more proximal amputation, however, typically results in fibrotic healing with minimal regeneration [1,10]. Initially, it was hypothesized that this regenerative capacity in mammalian digits was derived from an undifferentiated pluripotent cell population, called the blastema, which form via the de-differentiation of mature cells [1,9]. This hypothesis has since evolved, as our group and others have shown that mammalian digit regeneration is largely driven by lineage-restricted tissue resident cells [9,11], including a recent single-cell RNA sequencing analysis of the digit tip blastema [12]. Interestingly, a study involving lineage tracing and single-cell analysis demonstrated that the environment of the regenerating digit, rather than cell intrinsic factors, determines the ability to de-differentiate in a lineage-restricted manner [13].

This highlights the need to better understand the molecular mechanisms that underlie this naturally occurring regeneration in mammalian digit tips. Epidermal Wnt and nail stem cells have been shown to play a role in mouse digit tip regeneration [14,15,16], but the processes governing epidermal–mesenchymal interactions have not been characterized. Unraveling the key molecular mediators that activate and mobilize the tissue-resident stem cells could provide new targets for limb tissue repair. The mouse model of digit tip injuries provides a promising avenue to study the key cellular and molecular mediators of limb and digit regeneration [9,17]. The regenerative capacity of murine digits is dependent upon the level of amputation: successful regeneration occurs when at least 60% of the distal phalanx remains after amputation and does not occur if less than 60% of the distal phalanx remains [1,10,18], although a recent study demonstrated that there is still a limited endosteal blastema formation even in a more proximal distal phalanx amputation [19]. A comparison of the transcriptional changes in the cells that populate the pro-regenerative versus pro-fibrotic wound beds after digit amputations may identify key molecular signaling pathways involved in regenerative healing. 

In this study, we employed bulk DNA microarray analysis to compare the transcriptional profiles of cells from pro-regenerative and pro-fibrotic wounds in a mouse digit tip amputation injury model. We identified molecular targets consistent with the published literature and further evaluated them in vivo using adenovirus administration to overexpress and knockout genes of interest to confirm their expression. Finally, we used reporter mice to delineate the germ-layer restricted pattern of expression of these genes, revealing a role for hedgehog (HH) signaling in epidermal–mesenchymal interaction during digit tip regeneration. 

## 2. Materials and Methods

### 2.1. Animal Surgeries

All experiments were performed in accordance with Stanford University Institutional Animal Care and Use Committees and were approved by the Administrative Panel on Laboratory Animal Care at Stanford University (APLAC). All experiments were conducted in *n* = 5 mice between 12 and 16 weeks of age. The following mouse strains were obtained from the Jackson Laboratory (Bar Harbor, ME): C57BL/6J (wild-type), B6.129(Cg)-*Gt(ROSA)26Sor^tm4(ACTB-tdTomato,-EGFP)Luo^*/J (mTmG), B6.129S6-*Shh^tm2(cre/ERT2)Cjt^*/J, *Gli1^tm3(cre/ERT2)Alj^*/J (Gli1-CreER^T2^), *Gli1^tm2Alj^*/J, and Tg(TCF/Lef1-lacZ)34Efu/J. Rainbow mice (ROSA26^VT2/GK3^) were provided as a gift from the Weissman Laboratory, Stanford University School of Medicine. Rainbow mice have been described previously [9] for clonal lineage tracing of individual cells. This reporter strain contains a multicolor Cre-dependent reporter construct in the ROSA locus (*R26*^VT2/GK3^) and was crossed with mice containing a tamoxifen-inducible Cre transgene under the Gli1 promoter (*Gli1*^CreER^). All animal surgeries were performed under inhalation anesthesia with isoflurane (Henry Schein Animal Health) at a concentration of 1–2% in oxygen at 3 L/min. Digit tip skin was disinfected with betadine solution followed by 70% ethanol three times. Digit tip injuries were created as previously described [20], with digit amputations carried out using an operating microscope. After injury, bleeding was stopped using styptic powder. 

### 2.2. Tamoxifen Injections

Tamoxifen (Sigma-Aldrich, Burlington, MA, USA) was prepared in corn oil at 20 mg/mL, and a 5 mg dose was injected intraperitoneally.

### 2.3. Digit Tip Amputation

Digit tip amputations were performed on digits 2–5 along the proximal third of the nail bed, as described previously [9]. All reporter mice were injected with 5 mg tamoxifen to activate single-cell fluorescent marking at the time of surgical amputation.

### 2.4. Adenovirus Treatment

The Ad*Dkk1*, Ad*Rspo1*, and control AdFc adenoviruses were systemically administered to wild type mice (*n* = 5 mice/group) via tail vein injection. The dose of adenovirus was 5 × 10^8^ pfu per mouse. Adenovirus transfection was confirmed by histologic analysis of the intestine.

### 2.5. Histology

For fixation, limb samples were placed in 2% (vol/vol) paraformaldehyde for 16 h at 4 °C and then prepared for embedding by soaking in 30% (vol/vol) sucrose in PBS at 4 °C for 24 h. Samples were removed from the sucrose solution and tissue blocks were prepared by embedding in Tissue Tek O.C.T (Sakura Finetek). Frozen blocks were mounted on a MicroM HM550 cryostat (MICROM International GmbH), and 8-μm-thick sections were transferred to Superfrost/Plus adhesive slides (Fisher).

For X-gal staining, the slides were rinsed with PBS and washed for 10 min in the same solution at 4 °C. They were then placed in detergent rinse (0.1 M sodium phosphate buffer pH 7.3, 2 mM MgCl2, 0.01% sodium deoxy-chlorate, 0.02% NP-40) for 10 min at 4 °C and stained overnight in the dark at 37 °C in staining solution (detergent rinse with 5 mM potassium ferricyanide, 5 mM potassium ferrocyanide, 1 mg/ml X-gal). Finally, the slides were gently rinsed in PBS and mounted in glycerol.

### 2.6. Microarray Analysis

Murine tissue samples were harvested from the digit wound sites at day 7 post-injury (*n* = 6 mice/group). The digit tip tissue was explanted and then processed for microarray analysis [21]. Murine RNA was isolated, labeled, and hybridized to the GPL1261 GeneChip according to the manufacturer’s protocols (Affymetrix, Santa Clara, CA, USA). Each gene in the microarray was represented by 20 oligonucleotide pairs, with each pair consisting of an oligonucleotide perfectly matched to the cDNA sequence and a second oligonucleotide containing a single base mismatch. Raw microarray data (sample intensity files) were processed using LIMMA normalization, and principal component analysis (PCA) was performed using GeneSpring GX 11.0 (Agilent Technologies, Santa Clara, CA, USA), and then evaluated using the Significance Analysis of Microarray (SAM) toolkit in Microsoft Excel 2007 (Microsoft, Redmond, WA, USA). By utilizing a set of gene-specific *t* tests, SAM is able to identify gene expression changes that are statistically significant. The analysis assigns a score to each individual gene, dependent on the expression change based on the standard deviation of the gene repeated measurements. Permutations of repeated measurements are used to determine the false discovery rate (FDR) for genes equivalent to chance. Only genes that had both an FDR of less than two and at least twofold expression difference were selected. Gene Set Enrichment Analysis (GSEA) was applied to detect non-random distributions of gene subsets, with probes ordered according to SAM test statistics. Hierarchical clustering was performed in MATLAB (Mathworks, Natick, MA, USA), and pathway networks were constructed using Ingenuity Pathways Analysis (Ingenuity Systems, Redwood City, CA, USA). Probe intensity values had an acceptable standard distribution in all samples, indicative of good sample quality, and probes that failed to amplify in the 20th percentile (low-quality samples) from at least one sample were removed.

### 2.7. Cell Culture

We isolated and cultured murine dermal fibroblasts from digit tips. Digit tips were collected from mice and isolated by mechanical and enzymatic digestion using previously published protocols for murine fibroblasts [22]. The cells were then cultured under standard conditions until passage three. After culture, cells were exposed to various concentrations of HH (50, 100, or 200 ng/mL) from R&D Systems (Minneapolis, MN, USA). Cellular proliferation was quantified using a BrdU ELISA proliferation assay (ab126556; Abcam, Cambridge, UK) (*n* = 12 per group). 

### 2.8. Imaging and Analysis

Laser scanning confocal microscopy was performed using a Leica WLL TCS SP8 Confocal Laser Scanning Microscope (Leica Microsystems) located in the Cell Sciences Imaging Facility (Stanford University, Stanford, CA, USA). The ×10, ×20, and ×40 objectives were used (×10 HC PL APO, air, N.A. 0.40; ×20 and ×40 HC PL APO IMM CORR CS2, H2O/Glycerol/oil, N.A. 0.75). Raw image stacks were imported into Fiji (Image-J, NIH) or Imaris (Bitplane) software for analysis. Fiji was used to make two-dimensional micrographs of the confocal data and to quantify fluorophore expression intensity. For analysis of clonality from rainbow mouse tissue, surfaces were created for each color of the rainbow construct expressed using the volume surface and thresholding tools in Imaris.

Fluorescent and bright-field images were taken with a Leica DM4000B microscope (Leica Microsystems) and RETIGA 2000R camera (QImaging Scientific Cameras).

### 2.9. Statistics

Statistical analysis was performed in Prism8 (GraphPad, San Diego, CA, USA) using one-way analysis of variance (ANOVA) with Tukey’s multiple comparisons test. Data are presented as means ± SEM. *p* values of *p* < 0.05 were considered statistically significant.

## 3. Results

**Figure 1:**
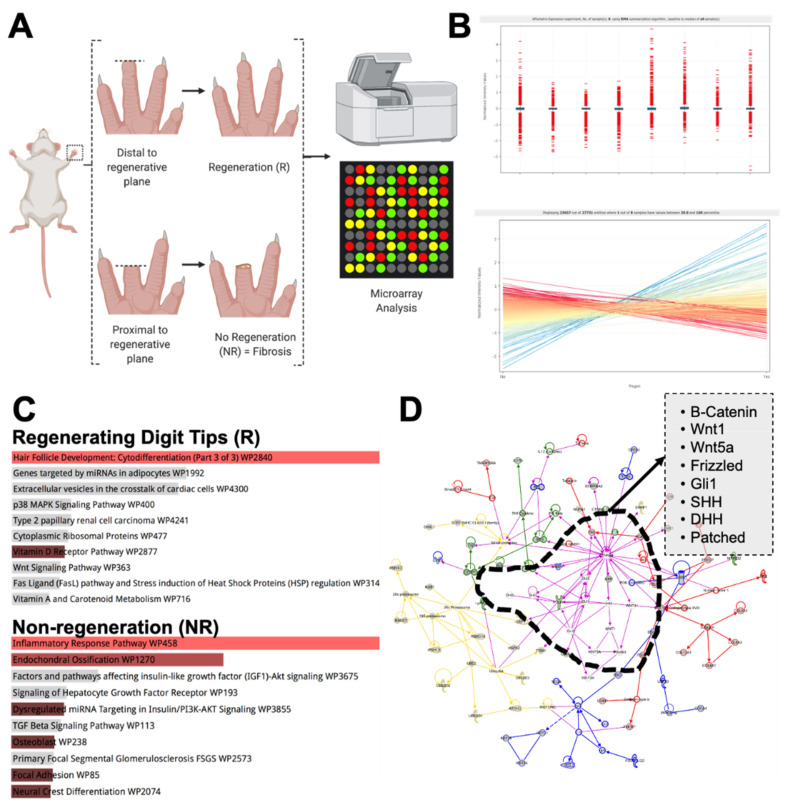
Microarray analysis of mouse digit tips. (**A**) Schematic of experimental conditions performed in *n* = 6 mice per group. (**B**) Quality control metrics demonstrate standardized data. (**C**) EnrichR enrichment pathway analysis of both regenerating and non-regenerating digit tips. (**D**) Ingenuity pathway analysis highlights most important and centralized genes for regenerating digit tips.

We amputated murine digit tips (*n* = 6 mice), either distal or proximal to the regenerative plane (Figure 1A) as previously described [9]. This allowed us to identify genes that were differentially expressed during regeneration while controlling for genes associated with normal wound healing. We found 287 genes that were differentially regulated in regenerating versus non-regenerating digit tips (Figure 1B, Supplementary Figure S1). From these genes, we chose to specifically investigate those that demonstrated at least a two-fold difference in expression.

**Figure 2:**
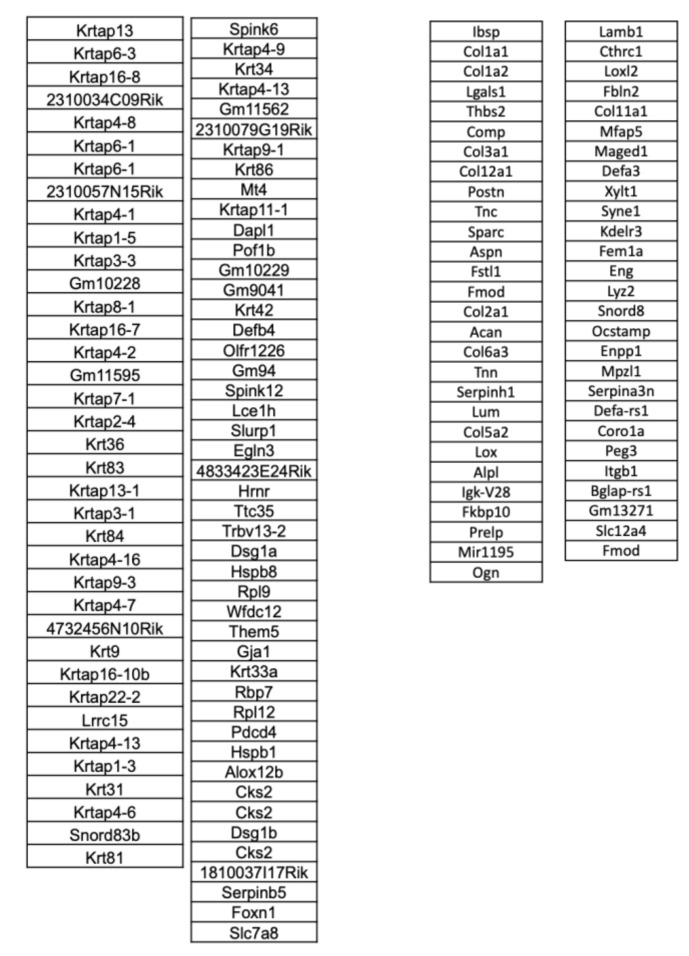
Top differential genes in regenerating digit tips and Top differential genes in non-regenerating digit tips.

We identified 84 genes that were upregulated (at least two-fold) in regenerating digit tips (Figure 2). This included a number of *Krtap* genes, which encode Keratin associated proteins associated with physiologic re-epithelialization and hair follicle development, as well as Wnt and HH signaling [23,24,25]. Gene set enrichment analysis (GSEA) using EnrichR [26,27] implicated hair follicle differentiation and confirmed the upregulation of the Wnt signaling pathway using the WikiPathway database (Figure 1C). We applied Ingenuity Pathway Analysis to the list of regenerative up-regulated genes, and we found that this pathway was centered directly around Wnt and HH signaling (Figure 1D). These genes have been previously linked to regenerative phenotypes, including both adipogenesis and hair follicle regeneration. Specifically, Wnt ligands have been found to be beneficial for mouse digit bone and nail epithelium regeneration [15,28], while HH signaling seems to stimulate hair follicle growth and drive dermal regrowth [29]. The presented microarray findings validate previously published reports identifying a significant role for Wnt in mouse digit tip regeneration [1,14,15].

A contrasting set of 55 genes were upregulated in non-regenerating digit tips. Specifically, many extracellular matrix genes were upregulated (*Col1a1, Col1a2, Col3a1, Col12a1, Col2a1, Col6a3*), indicating collagen production and fibrosis. These behaviors may be driven by myofibroblast differentiation and fibroblast proliferation, as shown by the upregulation of genes such as *Lgals1* and *Postn*, common in myofibroblast differentiation states [30]. Finally, these cells seemed to show genes related to chondrogenic differentiation, such as *Thbs2* and *Acan* [31]. Cells from the NR group also expressed *Lyz2*, a macrophage marker, potentially showing an increase in the number of inflammatory macrophages [32]. Indeed, when we performed the gene enrichment analysis, we observed increased pathways related to inflammation, endochondral ossification, and focal adhesion. These pathways are linked to increased collagen production, inflammation, and mechanotransduction, all of which we have previously classified as primary drivers of non-regenerative fibrotic healing [33,34,35]. 

**Figure 3:**
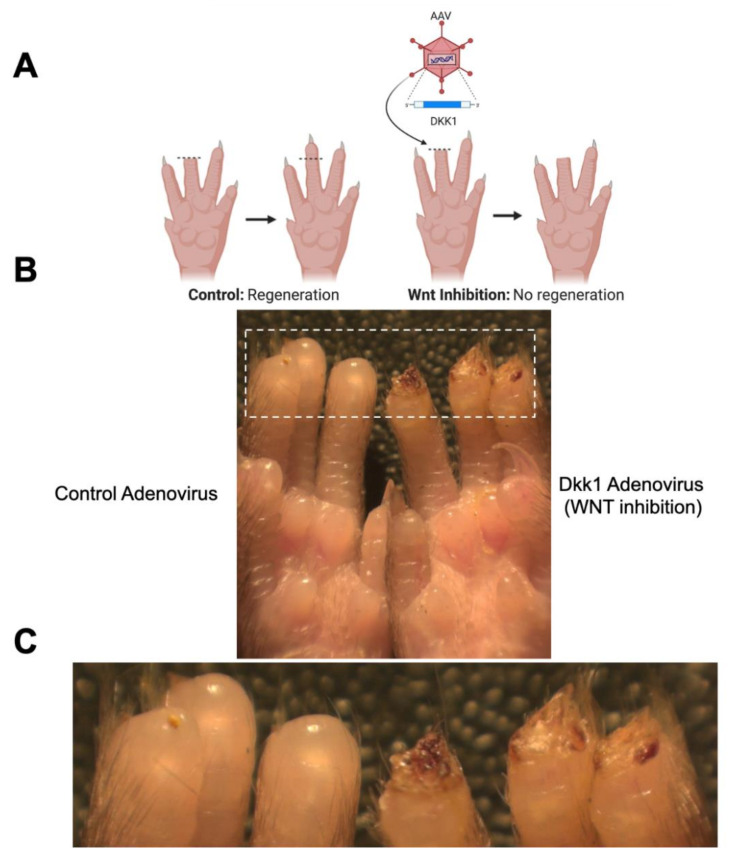
Wnt inhibition with Dkk1 adenovirus impairs epidermal closure during digit tip regeneration. (**A**) Schematic of experimental groups. (**B**) Picture of mouse paw. Mouse on left has not been treated; mouse on right has been treated with Dkk1 adenovirus. (**C**) Magnified image of (**B**).

**Figure 4:**
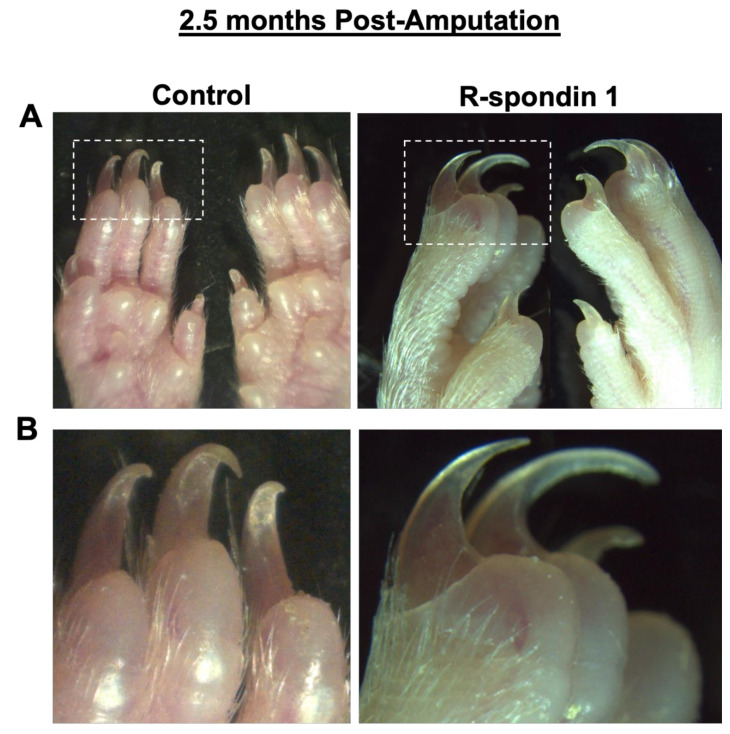
Upregulation of Wnt signaling with R-spondin adenovirus does not inhibit digit tip regeneration. (**A**) Amputated digits were found to regenerate normally compared to control. (**B**) Magnified image of (**A**).

To determine the functional significance and validity of our microarray data, we exposed C57BL6 wild-type mice (*n* = 5) to various adenovirus vectors. Mice exposed to *Dkk1*, a Wnt pathway inhibitor [36], demonstrated no evidence of regeneration when compared to mice exposed to a control adenovirus, in which the epidermis regenerated in 2–3 days and the nail regenerated in 7–10 days (Figure 3). The inhibition of Wnt expression reduced the regenerative capacity, consistent with our microarray data and previous studies [15,28]. We then examined the upregulation of Wnt signaling using an R-spondin adenovirus. R-spondin binds to Lgr5 receptors, neutralizing Rnf43 and Znrf3, two transmembrane E3 ligases that remove cell surface Wnt receptors [37]. The mice treated with R-spondin adenovirus were found to regenerate normally, with apparent nail overgrowth relative to control adenovirus-treated mice (Figure 4). Our findings are consistent with the literature demonstrating the critical role of Wnt during digit tip regeneration [15,28].

**Figure 5:**
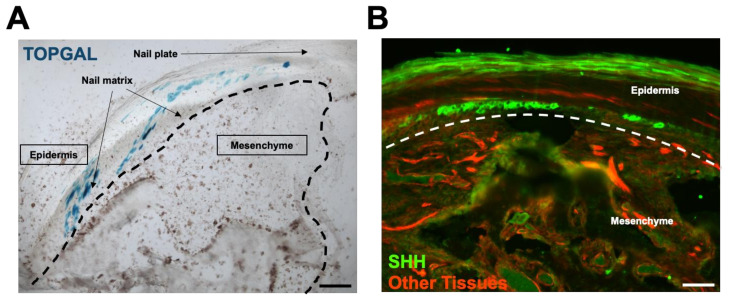
Wnt signaling and HH expression are restricted to epidermal structures. (**A**) TOPGAL reporter mouse identifies Wnt signaling. (**B**) **S**HH-CRE; R26 mTmG mouse identifies SHH expression pattern.

Having validated our microarray findings by suggesting a functional role for Wnt signaling during digit tip regeneration, we then sought to examine the spatial distribution and localization of Wnt and HH production and activity during regenerative events. Using a TOP-GAL reporter mouse, which expresses beta-galactosidase in response to activated beta-catenin, we found that, following amputation, Wnt signaling was restricted to epidermal structures, including the nail plate and nail bed (Figure 5A). This is consistent with and builds on the literature identifying Wnt expression in nail stem cells during digit tip regeneration [28].

Next, we crossed a transgenic mouse expressing Cre recombinase under the control of the SHH promoter with an R26 mTmG reporter mouse (SHH-Cre-ER: R26 mTmG). This construct resulted in SHH-producing cells expressing a green fluorophore, as opposed to the red fluorophore that was constitutively expressed in all other tissues. We found that, during digit tip regeneration, SHH expression, similar to Wnt signaling, was restricted to the epidermal structures, including the nail plate and nail bed, as indicated by the bright green staining (Figure 5B). 

**Figure 6:**
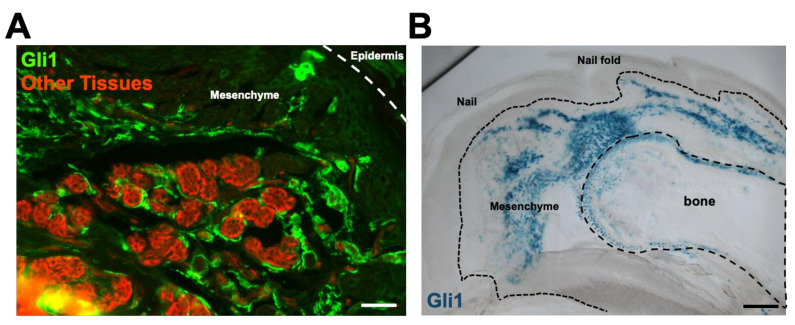
Hedghehog responsiveness is limited to the mesenchyme. (**A**) Gli1-CreER; R26 mTmG reporter mouse and (**B**) Gli1-lacZ reporter mouse demonstrate Gli1 expression, signifying canonical hedghehog signaling, is restricted to the mesenchyme.

We then used a transgenic Gli1 reporter mouse (Gli1-Cre-ER: R26 mTmG) to assess the HH responsiveness as Gli1 is a downstream effector in canonical HH signaling. Interestingly, whereas Wnt signaling and SHH expression are both restricted to epidermal structures, Gli1 expression, indicating HH responsiveness, was restricted to mesenchymal tissues, primarily in stromal fibroblasts and bone. These data indicate that canonical HH signaling originates in epidermal structures but exerts its effect in the mesenchyme during digit tip regeneration (Figure 6A). This was confirmed using a Gli1–lacZ mouse. Here, again, we see blue staining, indicating HH responsiveness within stromal fibroblasts and bone during digit tip regeneration (Figure 6B). These data suggest a previously unknown mechanism for epidermal–mesenchymal crosstalk during digit tip regeneration in adult mice.

**Figure 7:**
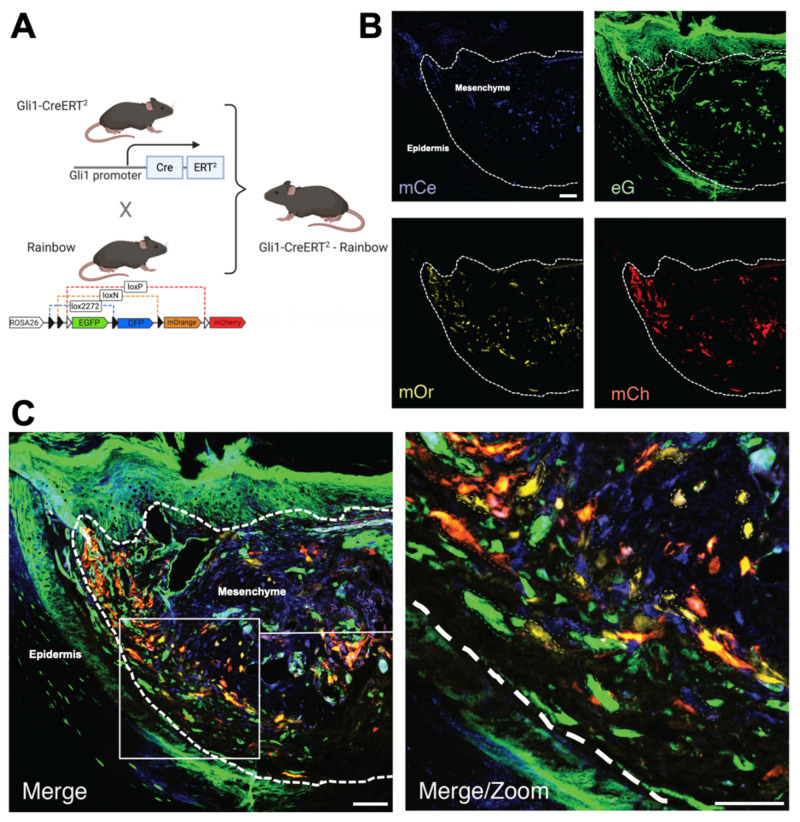
HH responsivene mesenchymal progenitors proliferate polyclonally during digit tip regeneration. (**A**) Schematic of mouse breeding. (**B**) Split channel images of digit tip after injury. (**C**) Merged and magnified views. Dashed lines outline clones.

**Figure 8:**
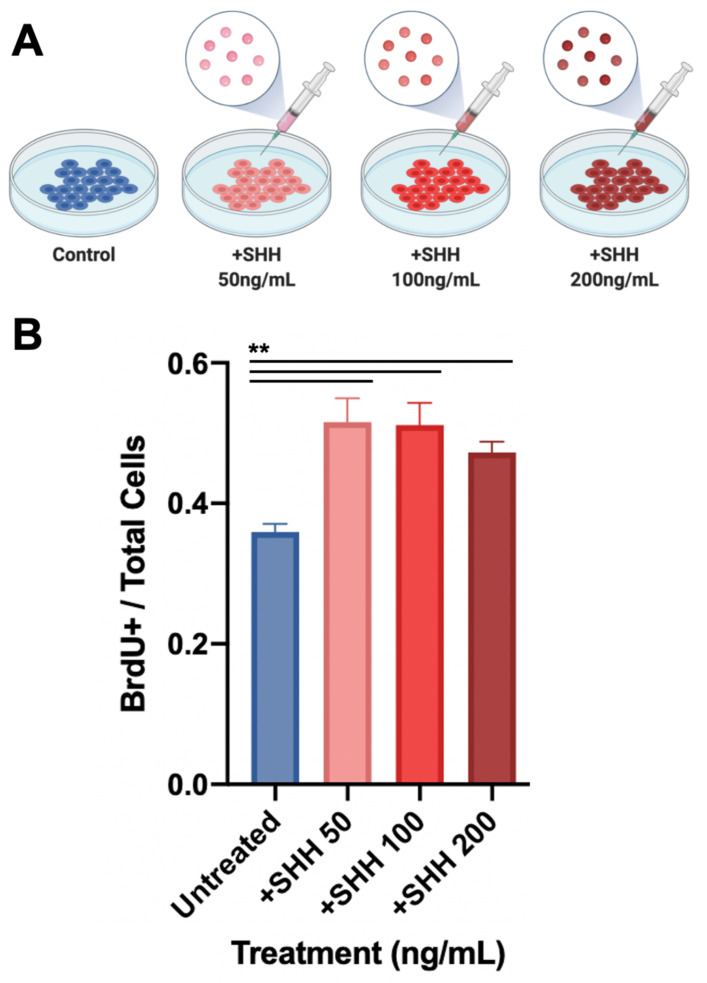
In vitro treatment with SHH increases fibroblast proliferation. (**A**) Schematic of experimental groups. (**B**) BrdU incorporation shows increased proliferation with SHH treatment at different concentrations. **: *p* < 0.001.

We have previously demonstrated lineage-restricted clonality during digit tip regeneration [9] using a rainbow reporter (*R26^VT2/GK3^*) mouse [38,39]. We wondered whether HH signaling may drive the clonality of mesenchymal tissue in the regenerating digit tip and reveal a functional role for the apparent epidermal/mesenchymal crosstalk. To answer this question, we crossed Gli1-CreER mice with rainbow mice to generate offspring, in which the tamoxifen-inducible, Cre-mediated recombination resulted in the stochastic expression of one of four different colored fluorophores (green fluorescent protein (GFP), cyan fluorescent protein (CFP), mOrange, and mCherry) in HH responsive cells and subsequent daughter cells (Figure 7A). A cluster of cells of the same color identifies the clonal expansion of a progenitor cell responsive to HH signaling during digit tip amputation. Two weeks following the amputation, we identified extensive clonal proliferation of HH-responsive mesenchymal cells within the regenerating digit tip (Figure 7B,C), suggesting that tissue-resident progenitor cells proliferate polyclonally in response to canonical hedgehog signaling during digit tip regeneration. To further evaluate this, we isolated murine digit tip stromal fibroblasts and cultured them. These cells were either left untreated or treated with 50, 100, or 200 ng/mL recombinant SHH (Figure 8). These specific concentrations were chosen based on previous research identifying significant SHH signaling at 100 ng/mL treatment in vitro [40]. Using a BrdU proliferation assay, we found that recombinant SHH significantly increased digit tip fibroblast proliferation at all concentrations, and by 42% at 50 and 100 ng/mL (*p* < 0.001). Taken together, these results suggest that epidermal-derived SHH stimulates the polyclonal proliferation of mesenchymal progenitors during digit tip regeneration.

## 4. Discussion 

Murine digit tip regeneration offers a compelling model to study mammalian regeneration. Amputation distal to the germinal matrix results in regeneration, whereas more proximal amputations result in non-regenerative fibrotic scars. Studies of digit tip regeneration in mice have demonstrated the role of lineage-restricted stem and progenitor cells and associated signaling pathways. However, the exact mechanisms that govern the complex interaction between various lineages during this process are still not fully understood. 

We sought to investigate this question in an unbiased manner to avoid relying solely on the existing literature and began with an exploratory microarray approach. This analysis revealed that non-regenerative fibrotic digit tip healing resulted from the elevated expression of collagen genes and genes associated with differentiation into myofibroblast and fibrotic phenotypes. These findings are consistent with the literature identifying these cells as the primary drivers of fibrosis as opposed to regeneration [35,41]. Our analysis of regenerative digit tips identified a central role for genes related to the Wnt and HH signaling pathways. The identification of Wnt by our microarray was consistent with recent studies that have demonstrated a critical role for epidermal-derived Wnt in mouse digit tip regeneration and underlying bone homeostasis [15,28]. For example, Takeo et al. identified nail stem cells that reside in the proximal nail matrix to govern differentiation and direct digit tip regeneration. They specifically used keratin 14 (K14 or Krt14) reporter mice to locate these nail stem cells and then *Axin2* reporter mice to examine the effect of Wnt. However, the mechanism linking these epidermal cells and the underlying mesenchyme was incompletely understood. We further validated our informatics analysis by assessing the functional significance of increased and decreased Wnt signaling and, as expected, Wnt inhibition impaired the ability to regenerate, while overexpression resulted in noticeable nail overgrowth. 

Next, we evaluated the spatial pattern of Wnt and HH signaling in the context of digit tip regeneration. Some recent evidence has indicated that Wnt and HH signaling are both related to hair follicle development through epidermal–mesenchymal interactions [29]. To explore these behaviors, we used transgenic reporter mice and observed that, while both Wnt signaling and SHH expression are limited to epidermal structures, downstream canonical HH signaling (through Gli1) occurs in the mesenchyme. This highlights Gli1-mediated HH signaling as a driver of epidermal–mesenchymal interaction during digit tip regeneration, which has not previously been demonstrated. To further explore this relationship, we used a hedgehog rainbow mouse, which demonstrated the polyclonal proliferation of HH responsive cells in the mesenchyme. Our in vitro studies reinforced this finding, demonstrating the increased proliferation of digit tip fibroblasts in response to treatment with recombinant SHH. 

It is possible that Wnt and HH directly interact after digit tip amputation to orchestrate the symphony of events necessary for the regenerative response. Time course studies to determine the dynamic activation of these two signaling pathways could point to whether they regulate each other. For example, does epidermal Wnt signaling precede the epidermal expression of SHH and potentially initiate it? Does the proliferative response of the mesenchyme to hedgehog signaling inhibit Wnt-driven epidermal expansion to prevent unregulated epithelial proliferation?

## 5. Conclusions

Overall, Wnt and HH signaling play critical roles in digit tip regeneration, and, while Wnt is germ-layer restricted, HH signaling drives epidermal–mesenchymal interaction. In the context of the literature, our findings synergize with various studies that have detailed the role of the Wnt pathway during digit tip regeneration [1,15,20,28,29]. Our microarray analyses pointed toward a novel HH pathway, which we then examined with transgenic reporter mice and in vitro analysis. Through these findings, we identify a previously undescribed mechanism for epidermal–mesenchyme crosstalk during digit tip regeneration in adults, as opposed to the previously studied contexts of embryonic development [42,43]. Our findings point toward an apparent mechanism that may also initiate crosstalk between these two germ layers in adult mammals. We bring together these findings to indicate an organized cellular response after injury that relies on Wnt and HH to facilitate proper bone regeneration, epithelial closure, and hair follicle regrowth. Further investigation of both Wnt and HH in the context of germ-layer-specific expression could reveal a possible interplay between these pathways. A better understanding of this interplay could inform therapeutic strategies for hand injuries by orchestrating a similar cascade to initiate regeneration in more proximal injuries.

## Data Availability

Original data may be obtained by e-mail request to the corresponding author.

## Supplementary Materials

The following are available online at https://www.mdpi.com/article/10.3390/jcm10184261/s1, Figure S1: Additional quality control metrics of microarray data.
