# Supplementary figures and images for: Epidermal-Derived Hedgehog Signaling Drives Mesenchymal Proliferation during Digit Tip Regeneration
Source: J Clin Med. 2021 Sep 20;10(18):4261. doi: 10.3390/jcm10184261 (PMC8467649; doi:10.3390/jcm10184261)

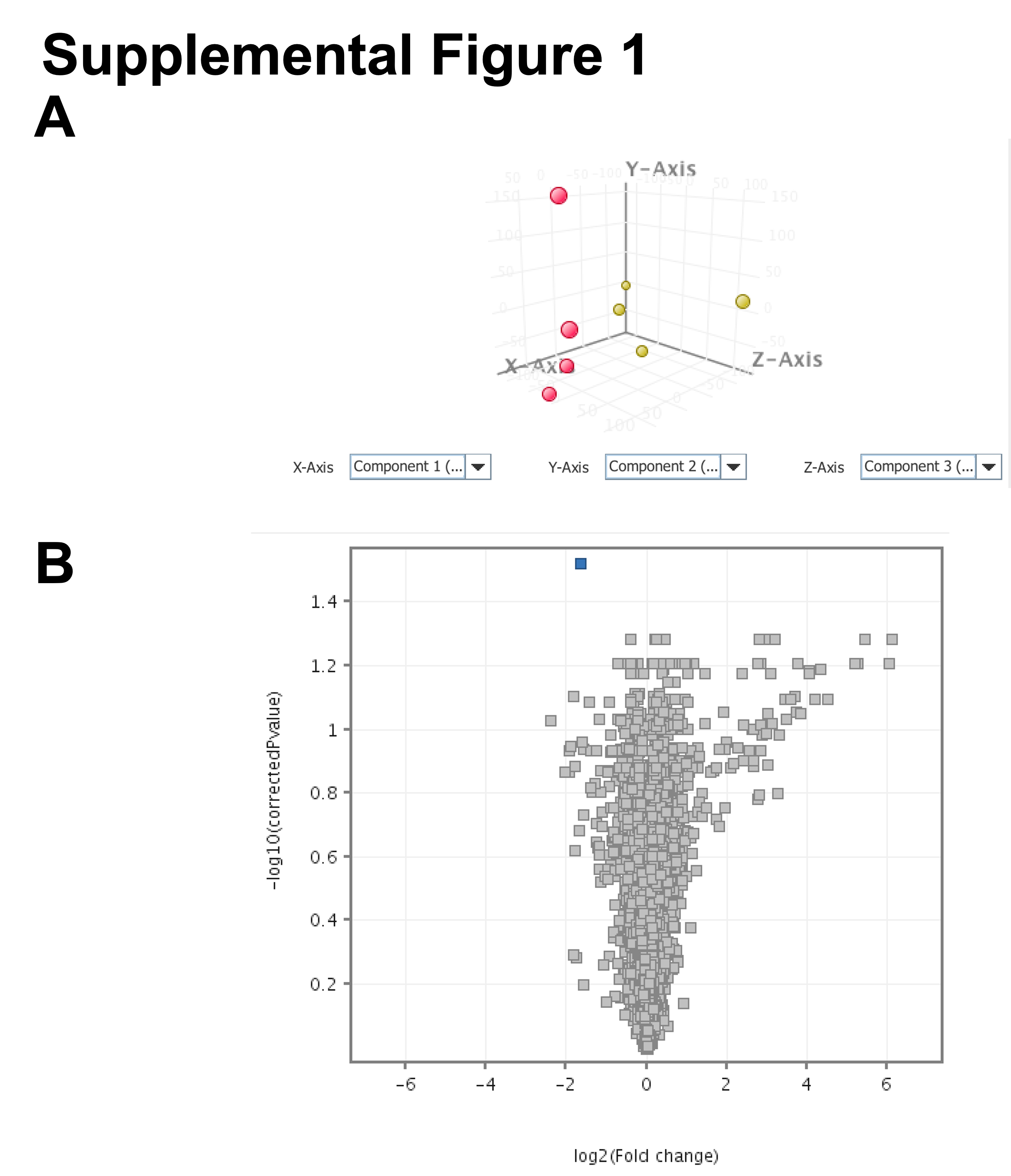

Supplement: Supplementary file 1 [file jcm-10-04261-s001.zip › jcm-1251157-supplementary.png]
